# Medical and Philosophical Intelligence

**Published:** 1813-09

**Authors:** 


(To be continued.)

---

### MEDICAL AND PHILOSOPHICAL INTELLIGENCE.

*ROYAL SOCIETY.*—On Thursday, May the 27th, a paper by Mr. Cater was read, comparing the Cassegrenian and Gregorian telescopes. These telescopes have been hitherto considered as quite similar. Mr. Cater was led to compare them in consequence of telescopes of both kinds being constructed by a self-taught artist at Ipswich, who has acquired the art of constructing both in remarkable perfection. The result of the comparison was, that the Cassegrenian telescope gave a much clearer and better defined image of the object than the Gregorian. Mr. Cater endeavours to account for this difference, by supposing that in the Gregorian telescope the particles of light interfere, and impede one another; while this does not happen in the Cassegrenian.

On

On Thursday, June the 3d, part of a paper by Mr. Brande was read, containing additional facts and observations on the use of magnesia in cases of urinary calculus. The paper was divided into two sections. In the first, Mr. Brande related several cases, in which the deposition of uric acid in urine and the accompanying symptoms were removed by the use of magnesia. Among others, a Gentleman of fifty-five was afflicted with pain in the kidney; a calculus at last passed into the bladder, and was voided by the urethra: it consisted of uric acid. His urine deposited a considerable quantity of red sand (uric acid). He tried alkalies, but they disagreed with his stomach. He was induced, in order to alleviate the symptoms of indigestion, to take a tea-spoonful of magnesia daily. His symptoms were gradually removed, and the urine ceased to deposit red sand. Several similar cases were given, in all of which the deposit of uric acid was put an end to by the use of magnesia. But in one case, after a certain time, the symptoms were aggravated, and a white sediment was deposited. This turned out, on examination, to be a mixture of phosphate of lime and phosphate of magnesia-and-ammonia. The object of the second section of the paper was to give an account of the treatment in such cases. Acids were administered, and different kinds were tried. Carbonic acid in the form of soda-water, citric acid, vinegar, cyder, oranges, lemonade, and muriatic acid, were tried in succession. The muriatic acid seemed to have answered worst. The other acids, in several cases related, removed the symptoms without inducing a deposition of uric acid.

On Thursday, the 17th June, Mr. Brande's paper was concluded. Another case was related, in which the deposition of the phosphates in the urine was put a stop to by the use of carbonic acid, produced by mixing lemon juice and carbonate of potash, and drinking the mixture while in a state of effervescence. From these cases Mr. Brande concludes,

1. That when the alkalies, from any circumstance, cannot be used to put a stop to the deposition of uric acid in the urine, then magnesia may be employed with advantage.

2. That the deposition of the phosphates may be put a stop to by the use of mineral acids.

3. That the vegetable acids produce a similar effect, and that they may be employed in much greater quantity, without injuring the digestive organs.

On the same evening a paper was read by Mr. Axlay, of Bristol, on the phenomena of electricity. He began by stating what he considered as his peculiar theory of electricity; but which does not appear to differ from that of Cavendish, unless the fourth proposition be considered as peculiar. The theory consisted of the following propositions —

1. A fluid exists called the electric fluid.

2. It is attracted by all matter, with a force inversely as some power of the distance.

3. Its particles repel each other. Hence it is elastic and compressible.

4. Electrics have a stronger affinity for it than non-electrics.

On Thursday, June the 24th, a paper by Sir Everard Home, Bart. was read, containing additional observations on the squalus maximus, or great shark. Two of these fishes were caught at Brighton last December, and one of them was brought to London. It was particularly examined by Sir Everard and Mr. Clift, and this paper stated the result of the new observations. The figure given by Sir Everard along with his former paper is correct, except that a fin between the anus and tail is wanting. The liver is very tender, and consists of six lobes. The gall duct is dilated at the extremity which enters the intestines, the object of which seems to be to prevent the bile from returning into the liver. There is no gall bladder. The heart is very powerfully muscular, and there is a particular muscle connected with the valves, which Sir Everard conceives intended to impel the blood more powerfully through the gills when the animal is at a great depth under water; for the pressure of the water will, in that case, impede the circulation in the gills. It occurred to Sir Everard that this impeded circulation might be compensated by water at great depths containing more oxygen gas in solution than near the surface; but water being taken up from the bottom of a deep well, and examined, was found to contafn no more than water at the surface. It may be remarked here, that this trial was scarcely of such a nature as to be decisive. Well water, however deep, is nothing else than rain water which has made its way through the earth to the bottom of the well; and all of it having been equally exposed to the air when falling in the state of rain, ought to contain the same portion of air. The water examined ought to have been taken from the bottom of the sea; but it is not likely that any perceptible difference would have been found. Indeed, the experiment has been already made by Biot, who examined sea water taken up at the depth of 437 fathoms, and found the proportion of oxygen gas in it the same as at the surface. (*Mem. d'Arcueil.* i. 273.) But the swimming bladder being filled with oxygen gas in those fishes that live at great depths, this in all probability is intended to answer some such purpose.

Sir Everard compared the heart of the squalus with that of several other animals. The squalus has no cerebrum, but only cerebellum. The cavity in which the semicircular canals of the ear is placed is uncommonly large. The lens of the eye is globular, and half sunk in the vitreous humour, which is very firm, and lodged, as usual, in separate cells. The retina is very thin. The cornea consists of three coats.—*Annals of Philosophy.*

---

*Linnæan Society.*—At the meetings of this Society on Tuesday the 1st, and Tuesday the 15th of June, a paper by Col. Hardwicke, on the bats in the British dominions in India, was read. He described and exhibited figures of 11 species, most of which inhabit trees, and live on fruits. The most remarkable of these is a very large species, having the aspect of a wolf, and of such a size that, from tip to tip, the wings extend 3 feet 8½ inches. This species the Colonel considers either as the vampyre of Linnæus, or as nearly allied to it. It lives on fruits, and is considered by the Indians as quite harmless.

The

The Colonel found that it would eat raw flesh when hungry; but that it preferred fruits. The stories of its sucking the blood of living animals he considers as quite unfounded.

The Society adjourned till the 2d of November.

---

IMPERIAL INSTITUTE OF FRANCE.

*Account of the Labors of the French Institute for* 1812.

(Continued from Vol. 29, p. 520.)

*Botany and Vegetable Physiology.*—Most physiologists have long admitted in plants an ascending sap, which mounts from the roots to the branches, and contributes to the increase of the branches in length; and a descending sap, which goes from the leaves to the roots, and to which some persons ascribe the chief part of the growth of the wood, and of course the increase of size in the trunk.

M. Feburier, a cultivator at Versailles, has endeavored to collect these two saps separately. For this purpose he bored a deep hole in the trunk of a tree, and fixed a bladder upon the inferior surface, so as to prevent any liquid coming from the lower part of the tree from making its escape into this hole. He made another hole in the tree, and placed a bladder in the same way against the upper surface. He considered the sap collected in the lower bladder as the ascending sap, and that collected in the upper bladder as the descending sap. He gives many observations on the relative proportions of each in different circumstances. Wishing, in the next place, to determine the route which each of these saps takes in the inside of the tree, he plunged alternately by the two ends branches of trees in colored infusions. In both cases these infusions appeared to him to follow the woody fibres surrounding the pith. This induces him to ascribe the same route to both saps, in which respect he coincides with the result of other experiments made by Mustel.

M. Feburier thinks, likewise, that the ascending sap contributes principally to the growth of the branches, the descending to that of the roots. But he thinks that the *cambium*, or that humor which transudes horizontally from the trunk, and which is looked upon as the matter which occasions the increase of the tree in thickness, proceeds, like the peculiar juices, from the mixture of the two saps. The presence of the leaves, necessary for the production of the descending sap, is of consequence also for the increase of the plant in thickness. But the buds, which M. du Petite Thouars conceives to act so important a part in that operation, have nothing to do with it, in the opinion of M. Feburier; for it takes place, says he, as long as the leaves exist, and it ceases as soon as they are removed, whether the buds be left or not.

As far as concerns the flowers and the fruits, M. Feburier assures us that he has observed the ascending sap, when it predominated, tending to determine the production of simple flowers, and the complete developement of the germs; that the descending sap, on the contrary, when too abundant, occasions the multiplication of flowers and petals, and the growth of the pericarp, and by consequence of the fleshy fleshy part of the fruit; principles from which it would be easy to deduce many useful practices in gardening, and which explain various practices already pointed out by experience.

According to M. Feburier, the alburnum deprived of its bark, but kept from the contact of the air, is capable of reproducing, by means of the cambium, the bark and epidermis necessary to cover it, as the bark produces constantly, even when partly separated from the trunk, liber and alburnum. In this point he has for antagonist our colleague M. Palisot de Beauvois, who has likewise employed himself in the investigation of these difficult questions, respecting the direction of the sap, and the formation of wood. According to this botanist, this escape of a glossy humor, which some physiologists suppose to proceed from the old alburnum, and to contribute to the formation of the liber, is not founded on convincing experiments. On the contrary, when a portion of bark is removed from a tree, and the wound is well rubbed, so as neither to leave liber nor cambium, neither the alburnum nor the wood produce any thing; but the edges of the bark gradually extend themselves, cover the naked wood, and produce liber and alburnum incontestibly proceeding from that bark. M. de Beauvois announces that he will soon elucidate this proposition at full length, which hitherto he has only noticed incidentally in his Dissertation on the Pith of Vegetables.

The opinion of physiologists has been hitherto very much divided about the functions of the pith of vegetables. According to some, that organ is necessary to the life of plants during the whole of their existence: according to others, it is only useful during the first years of their life, or all the time that it is green and succulent, and may be easily confounded with the cellular texture. M. de Beauvois has made on this subject observations which tend to prove that the pith performs functions during the whole life of the plant, if not absolutely necessary to its existence, at least very important for its progress, and the growth of its branches, leaves, and especially of the organs necessary for its reproduction.

He has remarked that the circular layer, or fibres which immediately surround the pith, has always a form corresponding to the arrangement and disposition of the branches, twigs, and leaves: that in plants with verticillated twigs and leaves, for example, the horizontal section of this case of the pith shows as many angles as there are twigs at each stage, and at each verticilla.

In like manner, the medullary case of the laurel rose presents an equilateral triangle, if the branch below the verticilla has three twigs and three leaves; but if we cut it under the lower verticilla, where one of the twigs and leaves is usually wanting, it has only two angles, and the vestige of a third. This law is constant, even in herbaceous plants.

M. de Beauvois has begun similar observations on plants, with opposite, alternate, dichotomous, spiral, and pinnated leaves. He expects to find them in the same relation between the medullary case, and the disposition of the branches, twigs, and leaves. For example, opposite leaves seem to require a round medullary case, becoming oval, oval, and having its extremities more and more pointed the nearer it approaches to the insertion of the branches and the leaves. When the leaves are alternate, the circle is less perfect. The extremities become equally narrow, but alternately, and each on the side where the branch is to appear. When the leaves are spiral, the number of angles of the medullary case is equal to that of the leaves of which the spirals are composed. Thus the medullary case of the lime has four angles; that of the oak, the chesnut, the bramble, the pear-tree, almost all the fruit-trees, &c. has five angles, more or less regular, because the spirals succeed constantly in fives.

Grew and Bonnet alone seem to have been on the way of these observations. The first had observed very various forms in the medullary case, especially in that of the conical roots of culinary plants; but he did not observe the relation between these forms, and the dispositions of the branches and leaves. The second carefully examined vegetables with opposite, verticillated, alternate, and spiral leaves; but did not observe the connection of these dispositions with the form of the medullary case.

M. de Mirbel has continued his researches into the structure of the organs of fructification in vegetables, in which he has been seconded with a zeal and intelligence that he takes a pleasure in acknowledging, by M. Schubert, whom the Grand Duchy of Warsovia sent into France to complete his knowledge of botany, which he was afterwards to teach in Poland. These two botanists have examined all the genera of trees with needle-shaped leaves, or the coniferous, one of the most important to be known, on account of the singularity of its organization, of the greatness of the species which it includes, and of the utility of its products. Every body is able to distinguish, at the first glance of the eye, the cedar, the meleza, the spruce fir, the Scotch fir, the thuya, the cypress, the yew, the juniper; but though the botanists have studied with particular attention the organs of reproduction of these vegetables, they are not agreed about the characters of the female flower; or to speak more correctly, the greater number are of opinion that the stigmata of the spruce fir, the Scotch fir, the cedar, and the meleza, have not yet been observed. In this respect, therefore, these trees are in reality cryptogamous. MM. Mirbel and Schubert go farther: they affirm that the female flower of the yew, the juniper, the thuya, the cypress, &c. is not better known; and that all the genera of coniferous plants have a common character, which has hitherto misled observers, and which consists in the existence of a cup, not similar to that in the flower of the oak, but more deep, concealing entirely the ovarium, and contracted like the neck of a bottle at its orifice. The female flower enclosed in this case has escaped observation. In the thuya, yew, juniper, cypress, &c. the cup is strait; and by an error, occasioned by the extreme smallness of the organs, the orifice of this cup has been always mistaken for the stigma. In the cedar, meleza, spruce fir, and Scotch fir, the cup is reversed, and its orifice is very difficult to perceive. It is only within these few years that it has been observed in England by Mr. Salisbury, and in France by MM. Poiteau, Mirbel, and Schubert. These botanists have have not hesitated to consider it as the stigma; and this was natural, because it had been agreed upon that the stigma of the yew, the thuya, the cypress, &c. was placed at the orifice of the cup: but farther researches have undeceived MM. Mirbel and Schubert. By a delicate dissection, they have ascertained that what is generally taken for the female flower in coniferous plants is only the cup, the form of which resembles that of a pistil, and that it contains in its cavity the true flower, which is provided with a membranous calix, adhering to the ovarium, and with a stigma, sessile in all the genera except the *ephedra*.

It must be obvious that this structure, so different from what had hitherto been imagined, will occasion great changes in the explanation of the characters, of the family, and of the genera.

According to Mirbel, the female flower of the plants of the family of *cycas* has an organization analogous to that of coniferous plants. This supports the opinion of M. Richard, who places these two families together among dicotyledonous plants; but M. Mirbel is of opinion, that as long as the characters of vegetation serve for the base of the two great divisions of plants with visible flowers, the *cycadeæ* cannot be separated from the palms.

The organization of the male flower of mosses has likewise engaged the attention of Mirbel and Schubert. After Hedwig, it would have been difficult to discover any thing new on this subject; but the bursting of the anthers, and the emission of the pollen, were phenomena doubted of by several botanists. Our two botanists declare that they have seen the most unequivocal proofs of the existence of these phenomena. The organs which Hedwig calls male in the *Polytrichum commune*, placed upon water, split at the summit, and threw out an oleaginous liquid, which extended itself like a thin cloud on the surface of the water. Mirbel and Schubert then subjected to comparative observation the pollen of a great number of *phenerogamous* plants, and ascertained that they exhibited the same phenomena as the male organs of mosses. This induces them to think that the parts called anthers by Hedwig may be nothing else than simple grains of pollen of a particular shape.

M. Mirbel has continued his researches on germination. He has observed, contrary to the generally-received opinion, that the radicle does not always make its appearance first. In many cyperaceous plants it is always the plumula which appears first. The same botanist has republished, with important modifications and additions, his opinions respecting the organization of stems, respecting their growth, and respecting the structure, both internal and external, of the organs of fecundation of plants.

M. Henri de Cassini, son of one of our associates, a name so celebrated in astronomy, has presented to the Class a memoir, which augurs success in a different science. He has examined with peculiar care the style and stigma in the whole family of plants known by the name of *compound*, *syngenesious*, or *synantherous*; and these small organs have exhibited a number of curious differences, which have induced him to propose a division of these plants, founded solely on the modifications fications of these two parts of the pistil. We regret that we cannot follow the skilful observer in the details into which he has gone, and which he has described and drawn with remarkable neatness. It cannot be doubted but they will one day be of great service in perfecting the classification of this family, so numerous and so natural; the subdivision of which, in consequence, ought to be more difficult than of any other.

There are few families of vegetables so directly useful to man as the grasses, which comprehend wheat, rye, rice, mais, sorgho, sugar-cane, barley, oats, millet, &c. To name these plants is enough to show the importance of a work which would enable us to distinguish them with certainty. The characters hitherto employed are generally regarded as insufficient. At each step the observer finds himself stopped. It is difficult, indeed often impossible, to find the true genus of the plant which he examines. Frequently the characters adopted only apply to a few species, and do not occur in the rest of the genus. M. Palisot de Beauvois has undertaken a general examination of this family, which he has published under the name of *Essai d' Agrostographie.* He has endeavoured to put an end to all this confusion, and to give to each genus constant characters, easy to perceive, so that the observer can never be at a loss.

For this purpose he has been obliged to adopt new bases, which he has already announced in his *Flora of Oware and Benin.* These depend principally on the separation or union of the sexes, on the composition of the flower, and on the number of its envelopes. Twenty-five plates, in which all these characters are represented, facilitate the study of these plants, which interest all the orders of society, even those persons who do not make botany a peculiar study.

M. de Beauvois continues his *Flore d' Oware et de Benin,* the thirteenth number of which is published; and his *History of Insects* collected in Africa and America, the eighth number of which has appeared.

M. de la Billardiere has finished his collection of the rare plants of Syria and Libanus, by the fourth and fifth parts. The same naturalist has communicated to the Class peculiar and interesting observations in natural history, which he made during his voyage in the Levant, the publication of which has been interrupted by the longer and more dangerous voyage which he made in the Entrecasteaux, an account of which was given to the public several years ago.

M. Gouan, correspondent of the Class at Montpellier, has published a description of the generic characters of the *ginko biloba,* a singular tree of Japan, which has been long known in Europe, but which, never having blossomed, could not be properly classified in the system.

There is a family of plants much less important than the grasses in point of utility, but much more singular in their characters, and which can only be seen vegetating on the sea-shore, namely, the *fuci,* and marine plants analogous to them. M. Lamouroux, professor of natural history at Caen, placed favorably in a city so near the coast, has has made these plants a particular object of study. He gives them the common name of *thalassio-phytes*, and divides them into various tribes, the characters of which he has been obliged to take in all parts of the plant, because he could not find sufficient characters in the organs of fructification, which usually serve as a basis to these divisions.

---

*Calculus from the Urethra of a Hog.*—Dr. Thomson, in his Annals of Philosophy for July, mentions that he received some time ago from Mr. Colville, surgeon in Ayton, Berwickshire, a calculus extracted from the urethra of a hog. It is nearly spherical; weighs 44·2 grains; its specific gravity is 1·595; it is white; has a silky lustre; and is composed of a congeries of very small crystals, which, as far as can be judged by the eye, consist of flat four-sided prisms. The calculus is soft, so that the crystals are very easily separated from each other. This calculus is composed entirely of phosphate of lime. When heated with potash, no smell of ammonia can be perceived. The crystals dissolve without effervescence in muriatic acid, and are precipitated in the state of a white powder by pure ammonia.

---

*Hydrosulphurets.*—Thenard has lately published some observations on the hydrosulphurets, which deserve the attention of chemists.

1. When a saturated hydrosulphuret is heated along with sulphur, a portion of the sulphur is dissolved, and a quantity of sulphureted hydrogen gas escapes. If there be an excess of alkali, the sulphur is dissolved as usual, but little or no sulphureted hydrogen escapes. Hence we see that the hydrogureted sulphurets contain less sulphureted hydrogen than the hydrosulphurets.

2. When the saturated hydrosulphurets are raised to the boiling point, a portion of sulphureted hydrogen always makes its escape. By this method the hydrosulphuret of magnesia may be decomposed altogether, and hydrosulphuret of lime nearly so. Hydrosulphuret of potash and soda become very alkaline.

3. Hydrosulphuret of ammonia may be obtained in crystals by surrounding with ice a flagon containing a mixture of sulphureted hydrogen and ammoniacal gases. It crystallizes in needles, and is white; but becomes speedily yellow when exposed to the air. It is very volatile, rising spontaneously to the top of the phial in which it is kept. By this means it may be separated from the hydrogureted sulphuret of ammonia.

4. When ammoniacal gas and sulphur are passed together through a red-hot porcelain tube, hydrogen gas and azotic gas are disengaged, and a great quantity of hydrogureted sulphuret of ammonia crystallized. When this substance is put into a phial, pure crystals of hydrosulphuret of ammonia gradually sublime from it.

5. The fuming liquor of Boyle smokes in oxygen gas or common air; but not in azotic gas or hydrogen gas. Hence the smoking must be owing to the presence of oxygen. Thenard supposes that it acts by converting the liquor into hydrogureted sulphuret, or into sulphite.—*Ann. de Chim.* lxxxiii. 132.

To

*To Medical Practitioners in every Part of the United Kingdom.*

The Board of the National Vaccine Establishment have remarked, with great concern, that the mortality from the Small-pox has, this year, increased considerably. This effect they have reason to attribute to the inconsiderate manner in which great numbers of persons are still, every year, inoculated for the Small-pox, at public charities and private houses; and are, afterwards, required to attend two or three times a-week, at the places of inoculation, through every stage of the disease. The contagion, by these means, is propagated in an extensive manner, and to an alarming degree. The same observation has been made in Ireland. The Royal College of Surgeons of Dublin, in a late communication to the National Vaccine Establishment, observe, that " for several years, the Members and Licentiates of the College, and, it is believed, all regular Physicians and Apothecaries in Ireland, have adopted the practice of Vaccination, their confidence in the anti-variolous effects of which remains unshaken; but it has been ascertained, that some unauthorised practitioners continue to inoculate for the Small-pox, and thus renovate and support sources of contagion." To obviate this mischief, the National Vaccine Establishment most earnestly recommend to the several branches of the faculty, in every part of the kingdom, after the example of the most eminent members of the profession, both in London and Dublin, to discourage inoculation for the Small-pox, by an agreement among themselves to decline the use of variolous matter.* The Association of the Medical Gentlemen of Glocestershire, a copy of which is subjoined, is a model of good conduct, which, if imitated in all other parts of the kingdom, would soon open to us the pleasing prospect of the speedy extinction of a disease, which has, for centuries, been no less detrimental to the population of states, than prejudicial to the health of mankind. FR. MILMAN, President.

By Order of the Board, JAMES HERVEY, Reg.

*Resolutions of the Medical Practitioners in the County of Glocester.*

Resolved,—I. That we see, with regret, the prejudices hostile to Vaccination, which exist in this neighbourhood.

II. That the honor of our profession, and the reputation of this county, require that every possible means should be employed to dispel them.

III. That those gentlemen who are satisfied of the efficacy of Vaccination, be requested to unite with us in forming a Society, to be called "The Glocestershire Vaccine Association."

IV. That the objects of this Association shall be, to promote Cow-pox, and *discourage Small-pox Inoculation.*

V. That, with this intention, the members of this Association shall individually and collectively declare, that they, considering their knowledge of Cow-pox, *do not believe themselves entitled either to*

* We refer the reader to the Resolutions of the Royal Colleges of Surgeons of London and Dublin, published in the Report of the Board for 1812, in which their determination not to inoculate with variolous matter, is recorded.

*practice, or, in any way whatever, to sanction the use of Small-pox Inoculation,* and that henceforth they renounce it accordingly.*

| | |
|---|---|
| J. Baron, M.D. | |
| C. B. Trye, | |
| R. Fletcher, | |
| G. B. Drayton, | |
| C. Cooke, | Glocester. |
| J. Wilton, | |
| Jos. Mills, | |
| D. Cox, | |
| T. Washbourn, | |
| W. Washbourn, | |
| H. C. Boisragon, M.D. | |
| C. Parry, M.D. | |
| *T. Newell, | Cheltenham. |
| C. Seagar, | |
| W. Wood, | |
| *E. Humpage, | Painswick. |
| *S. Cambridge, | Minchinhampton. |
| T. Hughes, | |
| W. W. Darke, | Stroud. |
| *S. Snowden, M.D. | |
| *S. Humpage, | |
| W. Fry, | Dursley. |
| H. Jenner, | Berkeley. |
| J. C. Hands, | |
| *J. Terret, | |
| *W. Dillon, | Tewkesbury. |
| *W. S. Evans, | |
| *R. Lovesy, | Newent. |
| *R. Filkin, | N. Glocester Militia. |
| *O. W. Bartley, | Nailsworth. |
| *J. Cooper, | Wotton-under-Edge. |
| *T. Jennings, | Chepstow. |
| *T. Skey, | Sunday's Hill. |

33 Names.†

---

Resolved,—That this Board do unanimously approve the Resolutions formed at the meeting of the Surgeons and Apothecaries of Glocestershire; and do earnestly recommend them to be adopted by gentlemen of the profession in every part of the kingdom.

*February* 18, 1813. Fr. Milman, President.

---

* Unless in a case of *extreme* necessity; for example, the Small-pox breaking out among persons who never had that disease, where no vaccine matter can be obtained.

N.B. We are authorised to add the names of those gentlemen to which the asterisk is prefixed, though we have not yet had an opportunity of collecting their actual signatures.

† This number soon afterwards increased to sixty-three.

The Board of the National Vaccine Establishment having received intimation of an assertion "that vaccination protects people from the small-pox for a few years only," and being deeply impressed with the pernicious consequences which might attend the propagation of so rash an opinion, think it a duty they owe to the public to declare, from the most attentive and dispassionate consideration of the history of this disease, collected from every quarter of the world, that the assertion above-mentioned is wholly without foundation. That the security against the small-pox, where the insertion of the lymph has produced the vaccine disease, is permanent, and there is no space, as far as the lapse of time since its discovery will enable the Board to decide, which renders people who have evidently been vaccinated susceptible of the small-pox. The Board does not advance this proposition upon slight grounds, but upon the authority of actual experiment. The ingenious gentleman, Dr. Jenner, to whom the world is so much indebted, has tried in vain to communicate the small-pox by inoculation to persons who were vaccinated in 1796; and the same attempts have been made by other persons, with a similar result, on different people at various periods from the time of their vaccination. It is not hereby meant to be denied that small-pox has occurred after vaccination: on the contrary, it has been admitted, and the Board, in its last Report to Parliament,* has stated, on a calculation made in France on a large scale, that though it has happened, yet that the instances of its occurrence, in proportion to the number vaccinated, are very few, being as 1 to 381,666, and that the appearance of the small-pox a second time in persons who have had that disorder from inoculation, is more frequent. The surgeons appointed to the nine stations of this establishment in the metropolis have assured the Board, that they have never seen any of the persons who had been vaccinated by them attacked with the small-pox; and it is well known, that to the few persons to whom the small-pox has occurred in its most virulent form, not a single instance can be produced of its proving fatal. If, therefore, vaccination does not entirely prevent, it mitigates and disarms a dreadful disorder of its mischief, which, in former times, occasioned the death of one person, at least, in ten of those who were attacked with it.

(By Order of the Board) JAMES HERVEY, Register.
*Leicester-square, July,* 1813.

---

*Deaths by Small-pox from the London Bills.*

| | | | |
|---|---|---|---|
| May 11, | 13 | June 29, | 10 |
| 18, | 8 | July 6, | 11 |
| 25, | 7 | 13, | 6 |
| June 1, | 5 | 20, | 15 |
| 8, | 9 | 27, | 12 |
| 15, | 10 | August 3, | 38 |
| 22, | 5 | | |

* Printed by order of the House of Commons, May 1813.

 Community

*Community of Associated Apothecaries and Surgeon-Apothecaries of England and Wales.*—We have been favored, by W. T. Ward, Esq. secretary to the London Committee of Apothecaries and Surgeon-Apothecaries, with the interesting intelligence, that the above *Committee,* "having most assiduously sought and maturely considered every information they could collect, have agreed on and arranged certain *Resolutions,* which in their judgment appear best calculated to form the basis of a new Bill to be introduced into Parliament during the next session." These Resolutions, we are further informed, will be printed, and addressed to every corresponding member of the District Committees, by the third week in September; and, availing ourselves of this circumstance, we shall give them in the next number of this Journal. The publication of this intelligence affords the Committee the opportunity of respectfully requesting those gentlemen who have received, or promised subscriptions, to transmit to the treasurers all arrears, previous to the publication of the statement of the Funds, which will accompany the Resolutions.

---

A foul and wicked report has been circulated, that the plague actually existed in Wapping. This, however, has been fully disproved by the testimony of a medical gentleman, and the minister of the parish. Though we have at present escaped the visitation of this direful pestilence, and proper measures have been adopted to secure us from it, yet we ought not to rely too much upon the efficacy of our quarantine laws. It is true that a *lazaretto* is established off Ryde (Isle of Wight), and that persons having had communication with or coming from Malta are compelled to perform quarantine; and it is also true that some individuals have evaded the law on that head. With the plague raging so near us as at Malta, whilst so many of our countrymen are on that station, we should not consider ourselves as being fully secure; we should rather avail ourselves of the opportunity of obtaining all information upon the subject, that we may be prepared for the enemy, should he ever visit us. We hope some of our correspondents in the army or navy will shortly favor us with an account of the progress and effects of this fatal disease at Malta, as well as of the remedies employed for its extinction.

---

Mr. Singer has recently discovered that ammonia is spontaneously generated in some cases of electro-chemical decomposition, where nitrogen and hydrogen are slowly separated from nitric acid and water. The gradual evolution of its elements is essential in these experiments to the formation of the ammonia, but its appearance is distinct and satisfactory, and results from a very simple arrangement. Mr. Singer is extending his experiments on the subject, which is highly interesting from its connection with the general phenomena of voltaic decomposition. The only instance of the spontaneous formation of ammonia by art with which we were previously acquainted, is that furnished by Mr. W. Higgins, who produced it by the action of granulated tin on dilute nitric acid.

---

Sir Everard Home has in the press a course of Lectures on Comparative Anatomy, delivered by him at the College of Surgeons. The

The arrangement is intended to follow that of the Hunterian Museum; and will furnish an elaborate commentary on that admirable and unique collection.

Mr. Hodgson's Treatise on the Diseases of Arteries and Veins, comprising the Pathology and Treatment of Aneurisms and Wounded Arteries, will be published in October, in one volume octavo, illustrated by a series of engravings in quarto.

---

*Trinity College, Dublin.*—Lectures upon Anatomy, Physiology, and Surgery, by James Macartney, M.D. F.R.S. M.R.I.A. Professor of Anatomy and Surgery in the University, and late Lecturer upon Comparative Anatomy and Physiology, at St. Bartholomew's Hospital, London. These Lectures will be divided into three courses: 1. A preliminary one of general Physiology, which will be open to the public. 2. A strict course of Anatomy and Physiology, as applicable to medical science. 3. A course of Surgical Anatomy and Operative Surgery.

The Lectures will commence on the first Monday in November, and terminate at the end of the following April. Demonstrations will be given daily in the Dissecting-room, by Mr. Cross, member of the Royal College of Surgeons in London.

---

Dr. Merriman, physician-accoucheur to the Middlesex Hospital and the Westminster General Dispensary, will begin his course of Lectures on Midwifery and the Diseases of Women and Children, on Monday, October 11, at half past ten o'clock. The Lectures are read daily at the Middlesex Hospital. Students when properly qualified will be provided with patients to attend in labour.

---

Dr. Clarke and Mr. Clarke will begin their winter Course of Lectures on Midwifery and the Diseases of Women and Children on Monday, October 4th. The Lectures are read at the house of Mr. Clarke, No. 10, Upper John-street, Golden-square, from a quarter past ten o'clock till a quarter past eleven, for the convenience of students attending the hospitals.

---

*Theatre of Anatomy, Bartlett's-court, Holborn.*—Mr. John Taunton, F.A.S. Member of the Royal College of Surgeons of London, Surgeon to the City and Finsbury Dispensaries, City of London Truss Society, &c. will commence his autumnal Course of Lectures on Anatomy, Physiology, Pathology, and Surgery, on Saturday, October the 2d, 1813, at eight o'clock in the evening precisely, and they will be continued every Tuesday, Thursday, and Saturday, at the same hour.

In this Course of Lectures it is proposed to take a comprehensive view of the structure and economy of the living body, and to consider the causes, symptoms, nature, and *treatment of surgical diseases*, with the mode of performing the different surgical operations; forming a complete course of anatomical and physiological instruction for the medical or surgical student, the artist, the professional or private gentleman.

An ample field for professional edification will be afforded by the opportunity which pupils may have of attending the clinical and other practice of both the City and Finsbury Dispensaries.

---

*London Hospital.*—Dr. Buxton will commence his autumnal Course of Lectures on the Practice of Medicine, on Monday, October the 4th.

---

Dr. Clough, Physician-Accoucheur to the St. Mary-le-bone General Dispensary, and Endeavour Society, will commence his autumnal Course of Lectures on the Science and Practice of Midwifery, including the Diseases of Women and Children, as connected therewith, early in October next, at 68, Berner's-street, in the morning at half-past ten, and in the evening at seven; notice of which will be announced in most of the daily papers. Students, when properly qualified, will be amply provided with labours to attend in rotation, and their views forwarded by weekly examinations.

---

Mr. Carpue's Course of Lectures on Anatomy and Surgery commences on the 1st of October. For particulars inquire at his house in Dean-street, Soho.

---

Mr. Moor, Surgeon-Dentist to her Royal Highness the Duchess of York, will commence a Course of Lectures on the Structure and Diseases of the Teeth, on the 4th of November, in which will be explained the complete Practice of the Dentist. Further particulars may be known at his house, No. 6, Palsgrave-place, Temple.

---

Dr. Roget will commence his autumnal Course of Lectures on the Practice of Physic, at the Theatre of Anatomy, Great Windmill-street, on the first Monday in October.

---

Dr. Adams's autumnal Course of Lectures will commence at his house in Hatton Garden, on the first Tuesday in October, at twelve o'clock precisely.

---

Dr. Clutterbuck will begin his autumn Courses of Lectures on the Theory and Practice of Physic, Materia Medica, and Chemistry, on Monday, October the 4th, at ten o'clock in the morning.

---

*Theatre of Anatomy, Blenheim-street, Great Marlborough-street.*—The autumnal Course of Lectures on Anatomy, Physiology, and Surgery, will be commenced on Friday, the 1st of October, at two o'clock, by Mr. Brookes. Anatomical Converzationes will be held weekly. Spacious apartments, thoroughly ventilated, and replete with every convenience, are open all the morning for the purposes of dissecting and injecting, where Mr. Brookes attends to direct the students, and demonstrate the various parts as they appear on dissection.

---

Dr. Ramsbotham will commence his Lectures on the Science and Practice of Midwifery, on Monday, October 4th, at half-past five in the evening. Pupils have the advantage of a valuable and well-selected museum.

METEO-

